# Micro Methods for Megafauna: Novel Approaches to Late Quaternary Extinctions and Their Contributions to Faunal Conservation in the Anthropocene

**DOI:** 10.1093/biosci/biz105

**Published:** 2019-10-02

**Authors:** Jillian A Swift, Michael Bunce, Joe Dortch, Kristina Douglass, J Tyler Faith, James A Fellows Yates, Judith Field, Simon G Haberle, Eileen Jacob, Chris N Johnson, Emily Lindsey, Eline D Lorenzen, Julien Louys, Gifford Miller, Alexis M Mychajliw, Viviane Slon, Natalia A Villavicencio, Michael R Waters, Frido Welker, Rachel Wood, Michael Petraglia, Nicole Boivin, Patrick Roberts

**Affiliations:** 1 Department of Archaeology, Max Planck Institute for the Science of Human History, Jena, Germany; 2 Anthropology Department of Bernice Pauahi Bishop Museum, Honolulu, Hawai’i; 3 Trace and Environmental DNA Laboratory, School of Molecular and Life Sciences, Curtin University, Bentley, Western Australia, Australia; 4 Centre for Rock Art Research and Management, University of Western Australia, Perth, Australia; 5 Department of Anthropology and with the Institutes for Energy and the Environment, The Pennsylvania State University, State College, Pennsylvania; 6 Natural History Museum of Utah and with the Department of Anthropology, University of Utah, Salt Lake City, Utah; 7 Department of Archaeogenetics, Max Planck Institute for the Science of Human History, Jena, Germany; 8 School of Biological, Earth, and Environmental Science, University of New South Wales, Sydney, Australia; 9 College of Asia and the Pacific and the School of Culture, History, and Language, Australian National University, Canberra, Australia; 10 Australian Research Council Centre of Excellence, Australian Biodiversity and Heritage, Wollongong, New South Wales, Australia; 11 Research Laboratory for Archaeology and the History of Art, University of Oxford, Oxford, England; 12 School of Natural Sciences, University of Tasmania, Hobart, Australia; 13 La Brea Tar Pits and Museum, part of the Natural History Museum, Los Angeles County, Los Angeles, California; 14 Natural History Museum of Denmark, University of Copenhagen, Copenhagen, Denmark; 15 Australian Research Center for Human Evolution, Environmental Futures Research Institute, Griffith University, Brisbane, Queensland, Australia; 16 INSTAAR and Department of Geological Sciences, University of Colorado, Boulder; 17 Department of Evolutionary Genetics, Max Planck Institute for Evolutionary Anthropology, Leipzig, Germany; 18 Departamento de Ecología, in the Facultad de Ciencias Biológicas, Pontificia Universidad Católica de Chile, Santiago, Chile; 19 Instituto de Ecología and Biodiversidad, Santiago, Chile; 20 Center for the Study of the First Americans, the Department of Anthropology, Texas A&M University, College Station, Texas; 21 Evolutionary Genomics Section of the GLOBE Institute, University of Copenhagen, Copenhagen, Denmark, and with the Department of Human Evolution, Max Planck Institute for Evolutionary Anthropology, Leipzig, Germany; 22 Research School of Earth Sciences, Australian National University, Canberra, Australia

**Keywords:** megafauna, extinction, Anthropocene, interdisciplinary science, conservation

## Abstract

Drivers of Late Quaternary megafaunal extinctions are relevant to modern conservation policy in a world of growing human population density, climate change, and faunal decline. Traditional debates tend toward global solutions, blaming either dramatic climate change or dispersals of Homo sapiens to new regions. Inherent limitations to archaeological and paleontological data sets often require reliance on scant, poorly resolved lines of evidence. However, recent developments in scientific technologies allow for more local, context-specific approaches. In the present article, we highlight how developments in five such methodologies (radiocarbon approaches, stable isotope analysis, ancient DNA, ancient proteomics, microscopy) have helped drive detailed analysis of specific megafaunal species, their particular ecological settings, and responses to new competitors or predators, climate change, and other external phenomena. The detailed case studies of faunal community composition, extinction chronologies, and demographic trends enabled by these methods examine megafaunal extinctions at scales appropriate for practical understanding of threats against particular species in their habitats today.

The unfolding extinctions and resulting biodiversity crises that we observe today are likely a continuation of processes that extend far back into human prehistory. The Late Quaternary extinctions of megafauna (broadly defined as animals weighing over 45 kilograms or 100 pounds) have remained the subject of fierce debate in both archaeology and paleontology since the nineteenth century (Douglas 2006). Scholarly opinions tend to be broadly polarized between two camps: those who believe the primary cause of extinction to be the arrival of humans in new ecosystems, leading to overkill (Martin and Klein 1989) or habitat disruption (Saltré et al. 2016) and those who ascribe a more significant role to climatic fluctuations during the course of the last glacial (Wroe et al. 2013, Stuart and Lister 2014). Some recent work has set aside these one-size-fits-all explanations in favor of more localized studies with multicausal explanations for megafaunal disappearances (e.g., Villavicencio et al. 2016). Given the vast biological, ecological, and evolutionary diversity across the world's megafaunal species, there is little reason to assume that all taxa responded in the same way to human arrival or dramatic environmental changes (Lorenzen et al. 2011, Price et al. 2018, p. 25).

Examples of more context- and species-specific studies of Late Quaternary megafaunal population changes include Guthrie (2006), who demonstrated that some ­species, such as bison (Bison priscus), wapiti (Cervus canadensis), and moose (Alces alces), increased in abundance before and during Late Pleistocene human colonization of Alaska and Yukon Territory in North America. Meanwhile, the wild horse (Equus ferus) and mammoth (Mammuthus primigenius) declined in abundance—and in some cases, body size—prior to extirpation. Similarly, research in Asia (Louys et al. 2007, Roberts et al. 2014) and Africa (Faith 2014) has indicated that many megafauna survived the arrival or expansion of human populations and climatic variability during the Late Pleistocene. Such studies point to local extirpations and redistributions of species as a product of long-term environmental change, as well as a process that varied greatly between taxa and habitats. Nevertheless, these more nuanced perspectives remain exceptions within the context of over five decades of archaeological and paleontological investigations into megafaunal extinctions.

Although the more favored large-scale synthetic approaches have enormous potential to offer new insight into managing climatic instability and anthropogenic ecosystems, in many cases, the data may be inadequate for such ambitious comparisons. For example, in many regions the available chronometric information is largely outdated or poorly contextualized, with limited fossil data and species-specific ecological information. A recent commentary on these large-scale syntheses suggests that investigations of Late Quaternary megafaunal dynamics would benefit most through increased efforts toward robust fossil dating, detailed paleoecological analyses of specific taxa through time, higher resolution and well-contextualized paleoenvironmental proxy records, and refined studies of behavioral variance among human populations living alongside megafauna (Price et al. 2018). The development of new scientific methodologies makes it increasingly feasible to pursue such fine-grained investigations of megafaunal extinctions.

In this article, we aim to review the role of five such methods (radiocarbon dating, stable isotope analysis, ancient DNA, ancient proteins, and microscopy or high-­resolution imaging) in generating high-resolution data sets and facilitating context- and species-specific understandings of extinction chronologies, population dynamics, and paleoenvironments (see figure 1). For each method, we highlight case studies that demonstrate how the application of these methodologies has led to new, detailed understandings of the relationships among humans, megafauna, the climate, and the environment. Moving beyond the dichotomous assumption of humans or climate change as the ultimate culprit, these approaches engender compelling new questions, particularly in the context of species-level population biology and vulnerability, as well as ecosystem feedbacks. We argue that the data sets afforded by new laboratory methods not only enrich the detail with which archaeologists and paleontologists can approach processes of extinction and extirpation, but also allow studies of past megafauna to inform conservation science and environmental sustainability. Moreover, studies of this nature will increasingly enable larger-scale, synthetic efforts to draw broad lessons from human–megafauna interactions across space and time.

**Figure 1.**
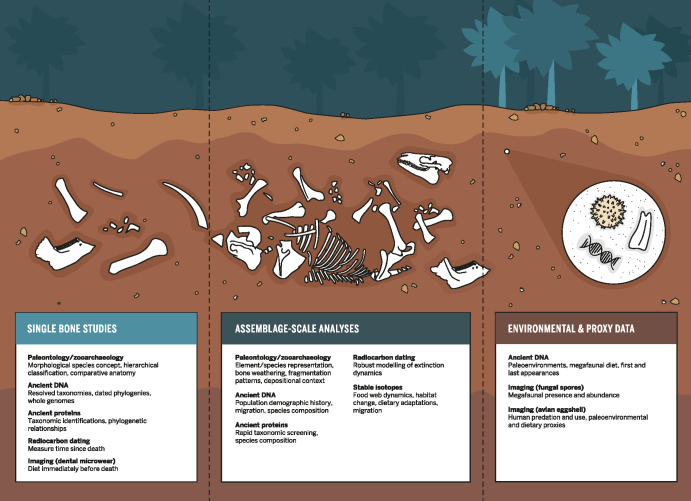
Advances in laboratory methods enable high-resolution insights into megafaunal extinctions. Applications of such methods (radiocarbon dating, stable isotope analysis, ancient DNA, ancient proteins, and microscopy or high-resolution imaging) complement traditional paleontological and zooarchaeological approaches to glean new insights via analysis of a single megafaunal bone, complete faunal assemblages, or the surrounding paleoenvironmental context.

## Radiocarbon methods

Radiocarbon dating can provide a direct measurement for the age of megafaunal specimens with preserved collagen. The advent of accelerator mass spectrometry (AMS) in the late 1970 s has enabled increasingly precise and accurate dates to be derived more quickly and from smaller sample sizes. These advances have helped uncover species-specific extinction trends in different parts of the world. For example, Orlova and colleagues (2004) reviewed the available radiocarbon dates for a variety of megafaunal taxa in Siberia. They demonstrated that although the final extinction of woolly rhinoceros and bison occurred around 11,000–9700 years ago, other taxa, such as woolly mammoth, horse, and muskox survived into the Late Holocene. Rigorous pretreatment protocols, such as ultrafiltration pretreatment, have also improved radiocarbon methods by efficiently removing contaminants from bone collagen. Samples contaminated with young carbon can lead to erroneous extensions of megafauna chronologies. For example, Higham and colleagues (2006) compared the effects of radiocarbon pretreatment methods on megafaunal remains from Kent's Cavern, the oldest site with evidence for Homo sapiens in the United Kingdom. They demonstrated that radiocarbon dates produced following the ultrafiltration approach can yield ages up to 7000 years older, as was the case for a woolly rhinoceros recovered from the site (Higham et al. 2006).

Targeting specific amino acids for radiocarbon dating promises to generate even more reliable chronologies for megafaunal extinctions. Radiocarbon dating of hydroxyproline, an amino acid that rarely occurs outside of bone collagen and is resistant to degradation, has been established as a gold standard for chronological accuracy (Devièse et al. 2018). This method has pushed back the local disappearance of western camels (Camelops hesternus) from Beringia to circa 50,000 years in the future, providing a new chronology that suggests this species’ disappearance is linked to changing climate and habitat restriction rather than the appearance of humans at circa 20,000 years ago (Zazula et al. 2017). However, the limitation of hydroxyproline dating is that it analyzes only a small fraction of datable material, and therefore can be untenable for smaller or more degraded samples. Dating multiple amino acids via the XAD-2 resin technique, which involves passing hydrolyzed collagen samples through a hydrophobic resin, overcomes this issue and is particularly effective at removing exogenous contaminants (Devièse et al. 2018). The application of this method to sites in Australia has called into question Holocene-age radiocarbon dates on megafauna, which had been used to argue for the late survival of certain megafaunal taxa alongside human occupation (Gillespie et al. 2015). Greater confidence in radiocarbon ages toward the chronological limit of this method enables more robust interpretations of the relationship between the last recorded identification of particular taxa to human, ecological, and climatic changes.

Bayesian radiocarbon modeling can also be a powerful tool for refining dating estimates. Bayesian models allow for the incorporation of relative (e.g., stratigraphic insights) and absolute (e.g., AMS radiocarbon dates) chronological information into a single statistical model. Such models can also be used to combine chronological records with other data sets, such as paleoclimate records. For example, Cooper and colleagues (2015) combined ancient DNA and detailed paleoclimate data alongside 31 radiocarbon-based time series to model extinction chronologies for a large number of megafaunal species in the Northern Hemisphere. They found a close relationship between rapid warming at the onset of the interstadials and regional extinctions or replacements of major genetic clades within megafaunal species. Such modeling exercises can only improve as additional high-precision radiocarbon data sets with appropriate quality assurance data are generated for study and fully published.

Although AMS radiocarbon dating methods remain the most precise in the context of the last 50,000 years, developments in radiogenic and luminescence dating methods have also improved megafaunal chronologies in contexts without organic preservation or extending beyond the limits of ^14^C. Moreover, Bayesian approaches can incorporate multiple chronological methods into the same model for greater confidence in evaluating the presence and disappearance of hominins and megafauna. For example, Douka and colleagues (2019) recently produced a chronometric model that combined radiocarbon, uranium series, and Optically Stimulated Luminescence ages, as well as stratigraphic and genetic data, in order to calculate precise ages for human fossils at Denisova Cave. Using this model they were able to estimate several key events: The range of Denisovan appearance at the site extended from around 195,000 years ago to circa 76,000–52,000 years ago. Meanwhile, Neanderthal appearances dated to 140,000–80,000 years ago. The arrival of the Upper Paleolithic, with bone tools and pendants, was constrained to 49,000–43,000 years (Douka et al. 2019). These methods could be extended to sequences where different megafaunal taxa, archaeological remains, and human fossils are present, and offers hitherto unattainable insights into human arrival, occupation, and megafaunal persistence or decline in different parts of the world.

## Isotopes and paleoecology

Stable isotope analysis of animal tissues has long been applied in ecology and archaeology to investigate environmental preferences, ecological niche partitioning, and dietary change. Reviews of the basic principles of stable carbon, nitrogen, oxygen, and strontium isotope analysis and their applications to paleoecology are widely available (e.g., Hobson 1999). However, it is only recently that such approaches have been used to study fine-grained details of megafaunal paleoecology. Stable carbon (δ^13^C), oxygen (δ^18^O), and nitrogen (δ^15^N) isotopes can now be used to derive significant physiological and ecological insights from analysis of even a single taxon. For example, Rawlence and colleagues (2016) used stable carbon and nitrogen from the bone collagen of individual moa species in New Zealand to tease apart differences in dietary and environmental preferences. By comparing this analysis with other dietary indices including coprolites and intestinal contents, they were able to investigate moa dietary preferences for different plant foods and plant parts, as well as environmental-versus-dietary influences on bone collagen stable isotope values. A similarly fine-grained data set was obtained by Larmon and colleagues (2019) via intensive sampling of stable carbon and oxygen isotopes from the dentine of a 27,000-year-old Eremotherium tooth from Belize. They were able to show how this individual varied its diet in response to seasonal fluctuations within a single year, granting high-resolution evidence for the ability of this species to adapt to climate-driven vegetation changes. The paleoecological resolution obtained by such methods enables new insights into the relationship of megafauna to their environments and how they might have been variously affected by climate change and human hunting activities.

In some cases, stable isotope analysis of megafaunal remains has enabled the reconstruction of Pleistocene food webs and ecological competition dynamics. For example, Fox-Dobbs and colleagues (2008) compiled δ^13^C and δ^15^N data from Late Pleistocene carnivores and prey species in the interior of eastern Beringia. Their results demonstrated dietary niche overlap and partitioning between different prey species. Horse, bison, yak, and mammoth seemingly relied on grasses, sedges, and herbaceous taxa, whereas caribou and woodland muskox focused on tundra lichen, fungi, and mosses. Data from carnivores indicated that all were generalist predators, apparently able to track and adapt to abundances of prey on the Beringian landscape, as well as to competition from other predators. This strategy may have contributed to the long-term persistence of certain species (e.g., wolves) and fluctuations in the populations of others (e.g., large felids and ursids).

This method can also place hominin species within a megafaunal ecosystem context. For example, δ^13^C and δ^15^N analysis of human and Neanderthal remains (Richards and Trinkaus 2009) alongside the remains of associated fauna have been used to argue for greater dietary diversity in humans contrasted by a top trophic level carnivore position for Neanderthals. However, a later study by Bocherens and colleagues (2014) observed δ^15^N shifts across an entire food web in southwestern France during this Middle to Upper Paleolithic transition. These shifts were large enough to also account for the observed δ^15^N differences between Neanderthals and humans, even without taking potential dietary differences into consideration. Such research highlights the importance of detailed, localized studies in which not only the species of interest but also their broader environmental context were considered. More recently, compound-specific approaches, which isolate and compare results from specific amino acids associated more closely with either local isotopic baselines (e.g., phenylalanine) or specimen trophic position (e.g., glutamic acid), have been used to support a top trophic level carnivore status for Neanderthals in France (Jaouen et al. 2019). Although these compound-specific approaches are still in development, such methods offer potential for more refined understanding of trophic positioning and the degree of pressure humans exerted on megafaunal taxa through hunting.

Stable isotope measurements of animal tissues can also be used to study climatic and environmental change experienced by different megafaunal species through time. For example, Graham and colleagues (2016) compared δ^15^N values from mammoth bone collagen from different temporal contexts on St. Paul Island, Alaska, alongside paleoenvironmental information from nearby lake sediment cores. They interpreted increasing δ^15^N in mammoth collagen, combined with increasing δ^18^O from lacustrine proxies, as markers for a period of declining precipitation that affected the plants consumed by mammoths in the build-up to their extirpation. Stable carbon and oxygen isotope ratios derived from inorganic tissues, such as tooth enamel, eggshell, and bone apatite carbonate, can also track paleoenvironmental change experienced by megafaunal individuals, usually across much longer time spans than δ^13^C and δ^15^N from organic tissues. In Australia, δ^13^C and δ^18^O analysis of a remarkable eggshell assemblage from extinct and extant giant birds, spanning 100,000 years and five regions across the continent, provides a long-term proxy for vegetation and climate change. δ^18^O values point to a period of relative climatic stability around the time of human arrival, and δ^13^C values suggest the permanent disappearance of C_4_ grasses on which some of these species apparently relied (Miller et al. 2016). Long-term paleoecological records such as these reveal environmental and ecological pressures on specific taxa, in contrast to off-site records that may have little relevance to the particular ecosystems under study.

Stable carbon, oxygen, and strontium (^87^S/^86^S) isotope ratios measured on sequentially growing megafaunal tissues can also track mobility and ranging, as well as the potential for climatic and anthropogenic factors to influence these behaviors. A study of wild horse and deer tooth enamel from Late Pleistocene Italy demonstrated that deer ranged more widely than wild horse (Pellegrini et al. 2008). However, both taxa remained within a relatively circumscribed area all year, which also provided human hunters with a stable (though vulnerable) resource. In Florida, comparison of mammoth tooth enamel ^87^S/^86^S suggested restricted megafaunal ranging (Hoppe 2004), contrary to previous theories that drew on modern elephant migrations to infer wide ranging in mammoths. Instead, mammoths may have been restricted to climatic refugia, which may have made them vulnerable to environmental change or human pressures. In eastern Australia, Price and colleagues (2017) analyzed a series of samples from a single, slow-growing incisor of Diprotodon optatum (the largest known marsupial) to infer seasonal migrations between regions with different isotopic signatures. Such megafaunal studies could, in turn, be linked to strontium isotope-based evidence for hunting ranges of past human (or other hominin) populations (e.g., Richards et al. 2008), and their potential impact and encounter rate with particular species on a landscape level.

## Ancient DNA

Ancient DNA (aDNA) has become a powerful tool for studying the human past, particularly with regard to population movements and interactions in prehistory. aDNA applications to animals have often been focused on processes of domestication and human-facilitated movements of different taxa from the Neolithic onward (e.g., Irving-Pease et al. 2019). However, a growing body of aDNA research on megafaunal taxa is furnishing new detail in the context of megafaunal species identifications and biodiversity. Although morphological interpretations of the fossil record suggested that only a single mammoth species roamed northeast Siberia during the Late Quaternary, aDNA from 25 dated mammoth samples from this region suggested the presence of two contemporaneous mammoth species (Gilbert et al. 2008). Similarly, mitochondrial genome analysis of Late Pleistocene mammoths from central Europe highlights the diversity and dynamics between mammoth populations across Eurasia (Fellows Yates et al. 2017). In contrast, morphological analysis of moa specimens from New Zealand has led to the definition of several different species of these flightless, megaherbivorous birds, whereas aDNA analysis has since demonstrated that some so-called distinct species were actually different sexes of the same taxon (Bunce et al. 2003). This taxonomic information provides the essential groundwork for more detailed studies of how megafaunal populations fluctuated through time and responded to various external pressures.

Developments in aDNA sequencing have now enabled the mapping of entire nuclear genomes of megafaunal fossils. The level of detail afforded by even a single genome can provide direct insights into the demographic history of generations past. Such information is often lacking in an area of study reliant on incomplete fossil records and chronologies. For example, by comparing the genome of one of the last surviving mammoths on Wrangel Island to an older mainland individual, Palkopoulou and colleagues (2015) were able to demonstrate that the Late Holocene Wrangel mammoth population was in steep decline and experienced high levels of inbreeding prior to extinction. A full nuclear genome from a Wrangel Island mammoth dating to around 4000 years ago also highlighted the accumulation of many mutations that potentially caused behavioral or developmental defects, which would have made these populations more vulnerable to extinction even in isolation. These inferred phenotypic properties included reduced male fertility, diabetes, and loss of the ability to detect floral scents (Fry et al. 2018). Genetically reconstructed population dynamics and functional traits can also be compared to climatic variation and human arrival. A collapse in Beringian steppe bison populations appears to be temporally correlated with climatic events that predate human arrival by millennia (Shapiro et al. 2004). Similarly, aDNA applications to cave bear and brown bear remains highlight the former's susceptibility to human predation due to restricted hibernation locations, as is evidenced by mitochondrial genetic lineages (Fortes et al. 2016).

Emergent molecular technologies are now enabling increasingly detailed studies of previously untapped paleontological resources. Bulk-bone metabarcoding (BBM) is a recently developed technique that allows for rapid aDNA analysis of entire fossil assemblages. The method takes hundreds of nondiagnostic bone fragments and grinds them together to make a synthetic bone mixture. Metabarcoding is then applied to elucidate the species composition of this mixture (Murray et al. 2013). BBM approaches are particularly well suited to assemblages of fragmented and morphologically unidentifiable zooarchaeological remains. Analysis of these materials can provide a broad picture of biodiversity prior to, during, and after climatic events or human arrival. For example, application of bulk bone metabarcoding in New Zealand identified 13 species of now-extinct endemic avifauna in archaeological and paleontological deposits, further characterizing faunal biodiversity in New Zealand before and after human arrival (Seersholm et al. 2018). This method could also be applied to search for appearances of megafauna in archaeological hearth or midden features, which would provide direct evidence for human hunting.

Molecular traces of megafaunal species can also preserve in sediments in the form of DNA from urine or feces. Analysis of these sediments can be used to recover DNA of megafaunal species and fine-tune extinction chronologies and biodiversity estimates. For example, aDNA of mammoth and horse from Alaskan sediments moved the North American extinction dates of both species forward several thousand years (Haile et al. 2009). More recently, targeted capture of mammalian mtDNA fragments from sediments demonstrated the presence of numerous extinct taxa in Late Pleistocene layers of archaeological cave sites in Eurasia (Slon et al. 2017). In another study, woolly mammoth DNA retrieved from sediment cores indicated an extinction date on St. Paul Island, Alaska that aligns closely with estimated dates from Preussia (formerly Sporormiella; Kruys and Wedin 2009) records (see below) and radiocarbon dates on fossils (Graham et al. 2016). Sedimentary aDNA also provides a window into the environmental context surrounding megafaunal extinctions. Analysis of the permafrost regions of the northern hemisphere documents profound vegetation shifts coincident with the last glacial–interglacial transition (Willerslev et al. 2014).

## Ancient proteins

Few ancient protein studies have as yet directly addressed questions concerning megafaunal extinction. However, recent publications show the potential roles for paleoproteomics in future megafaunal studies, especially given the longer-term preservation of protein molecules relative to DNA. Collagen peptide mass fingerprinting, also known as zooarchaeology by mass spectrometry (ZooMS), is a quick and efficient tool to taxonomically identify tissues rich in collagen type I (e.g., bone, dentine, skin, and antler) through the use of MALDI-TOF mass spectrometry (Buckley et al. 2009). Much like bulk bone aDNA metabarcoding, ZooMS enables large-scale screening of morphologically unidentifiable bones from Quaternary sites, in order to search for specific megafaunal taxa. However, unlike BBM, ZooMS is minimally destructive, and therefore additional analyses (e.g., radiocarbon dating, isotope analysis, and aDNA) can be conducted on a ZooMS-analyzed sample (see table 1 for a comparison of methods; e.g., Fellows Yates et al. 2017). The application of this rapid, cost-effective technique promises to drastically increase the quantity and diversity of megafaunal taxa identified in Late Quaternary sites across the world, improving confidence in analyses of change in regional faunas.

**Table 1.** Comparison of three biomolecular methods (bulk bone aDNA metabarcoding, sedimentary aDNA analysis, and collagen peptide-mass fingerprinting) for deriving community-scale taxonomic information without analyzing morphologically identifiable zooarchaeological remains.

	Bulk Bone aDNA Metabarcoding (BBM)	Collagen Peptide Mass Fingerprinting (ZooMS)	Sedimentary aDNA
Sample substrate	Entire fossil bone assemblages, including fragmentary and nondiagnostic elements	Single fragmentary, undiagnostic bone element	Sediment
Cost (per sample)	$40–$80 (excluding sequencing costs)	Under $10	$40 (excluding sequencing costs)
Processing time	2–4 weeks	2 days	4 weeks
Primary research product	In-depth insights into faunal assemblages	Taxonomic classification for individual specimens	Identifications of various floral and faunal taxa present in a sample
Additional by-products	Rapid assessment of DNA preservation through time; the ability to identify genetic haplotypes	Protein damage assessment for individual specimens	DNA damage assessment per taxon; taxonomic composition of microbial DNA
Considerations	Well suited to all vertebrates but especially valuable in identifying fish, reptiles and amphibians that are not easily identified using morphology; best suited when assemblages are compared through a sequence.	Currently largely restricted to mammals; limited phylogenetic depth; dependent on available peptide marker databases.	Authentication of the DNA fragments critical for interpretation; accuracy of taxonomic classification dependent on available comparative databases; movement of DNA across layers needs to be considered.
Replication	Can do replicate bone samples to check for species saturation. If entire bone fragment is used additional studies on same specimen are not possible.	Can conduct multiple and subsequently alternative analyses on specimens of high taxonomic interest.	Can conduct multiple and subsequently alternative analyses on samples of interest.
Ideal contexts	Ideally suited to cold or temperate localities but has been applied to warm tropical environs. Tropical environments require short metabarcoding assays.	Proteins are accessible from permafrost to tropical localities.	aDNA preserves better in cool, stable environments. Best suited to well-stratified deposits.

ZooMS assemblage screening studies can also provide insights into faunal community structure and biodiversity. For example, ZooMS analysis of a fragmented component of the Châtelperronian bone assemblage at Les Cottés, France identified almost double the number of species than those recorded via morphological analysis of better-preserved bones, despite the smaller size of the ZooMS assemblage. These additional identifications significantly increased the taxonomic richness of the overall assemblage, providing a more thorough interpretation of faunal community structure at Les Cottés (Welker et al. 2015a).

In addition, ZooMS can be used to identify ancient human remains, which could refine chronologies for the dispersal of Homo sapiens and inform on degrees of chronological overlap with extinct megafauna. ZooMS has so far been extremely effective in refining the geographic extent and chronology of Neanderthals up to their final extinction in Europe, as well as their potential overlap with modern humans. For example, Brown and colleagues (2016) applied collagen peptide-mass fingerprinting to an assemblage of over 2000 bones from Denisova Cave, Russia as a rapid-screening method for the discovery of hominin remains. Through this method, they discovered the Denisova 11 bone, which genome sequencing later determined belonged to a first-generation offspring of a Denisovan father and Neanderthal mother (Slon et al. 2018).

Protein sequencing can also clarify taxonomic identifications and phylogenetic relationships in the absence of aDNA preservation. For example, Welker and colleagues (2015b) sequenced the type I collagen α1- and α2-chains of the Late Quaternary South American native ungulate taxa Toxodon (Notoungulata) and Macrauchenia (Litopterna). By comparing these results with available collagen (I) gene transcripts from mammalian genomes as well as mass spectrometry-derived sequence data, they generated a phylogenetic tree that placed these taxa in a monophyletic group most closely related to Perissodactyla (Welker et al. 2015b). More detailed protein sequencing using liquid chromatography-tandem mass spectrometry (LC-MS/MS) has also enabled the phylogenetic placement of Late Pleistocene woolly rhinoceros, as well as elucidated relationships between extinct and extant taxa (Welker et al. 2016). The extinct taxon (Stephanorhinus) was was shown to be most closely related to the genera Coelodonta and Dicerorhinus. Meanwhile, the Sumatran rhino was grouped together with the genus Rhinoceros, rather than in the same clade as black and white rhinoceros species. Given the possibility of extracting phylogenetically informative protein sequences preserved within tooth enamel (Cappellini et al. 2018), paleoproteomic applications to the study of megafaunal taxonomy, biodiversity, and extinction is likely to significantly increase in the future.

## Microscopy and high-resolution imaging

Microscopy and other high-resolution imaging techniques can build on more traditional morphological analyses and provide both direct and proxy evidence for human–­megafauna interactions in the past. A particular point of contention in many regions is the degree to which humans and megafauna populations interacted. Putative butchery marks have frequently been questioned because of a lack of detailed taphonomic research or robust indicators, and variable interpretations by different analysts (Blumenschine et al. 1996). However, in-depth taphonomic studies of faunal assemblages have increasingly made use of advances in microscopy and high-resolution imaging to assess anthropogenic and nonanthropogenic surface modification. For example, Karr (2015) reviewed the taphonomic evidence for human–megafauna interaction at the Late Pleistocene sites of Owl Cave in Idaho and Inglewood in Maryland. By combining high-resolution imaging, experimental research, and in-depth consideration of human and environmental influences on bone taphonomy, Karr deduced that a variety of nonhuman factors likely contributed to the taphonomic patterns present at both sites, and concluded that it was not possible to definitively correlate the available taphonomic evidence with direct human–megafaunal interaction (Karr 2015).

Advances in confocal microscopy give significant insights into megafaunal diet and ecology. In particular, dental microwear texture analysis (DMTA) uses white-light confocal profilometry and scale-sensitive fractal analysis to quantify dental microwear, characterizing overall surface textures in 3-D and distinguishing consumption of hard, soft, and tough objects. Combined with stable isotope analysis, this approach has identified the dietary niche of the giant macropod Procoptodon, providing important context for its eventual extinction (de Santis et al. 2017).

Innovative use of microscopy techniques can also improve our understanding of human–bird interactions, an understudied component of past human–animal dynamics, particularly in the case of avian megafauna. For example, scanning electron microscopy (SEM) has successfully been applied to the study of archaeological eggshell to evaluate whether ancient communities in the American Southwest were practicing intentional husbandry of turkey populations versus hunting wild birds (Beacham and Durand 2007). This study relied on eggshell microstructural features that correspond to the ontogenetic age of the chick when the egg was broken (Chien et al. 2009). The application of SEM revealed whether eggshell in archaeological deposits derived from fully developed eggs that hatched naturally, a signal that people were raising turkeys, or from eggs that were prematurely broken, which indicated hunting of wild birds. Others have begun to outline approaches to ootaxonomy on the basis of variation in eggshell microstructural features. Although attempts at identifying bird species from fragmentary eggshell remains a challenge (Buss and Keiss 2009), further efforts to describe interspecific differences in eggshell morphology will improve our ability to use this technique. These approaches to eggshell microstructural analysis are relevant to studies of extinct giant flightless birds, including New Zealand's moa (Dinornithiformes) and Madagascar's elephant birds (Aepyornithidae). As ground-dwelling birds, these megafauna may have been particularly vulnerable to human activities, including hunting of adult individuals and predation on eggs (Miller et al. 2016). Moreover, improved ootaxonomic identification may reveal unique extinction trajectories of individual species within now-extinct avian families. Several imaging modalities, including SEM, computed tomography, and optical profilometry, can be used to study eggshell microstructural features and further elucidate the processes that led to extinctions of many of the world's giant avifauna.

Modern distribution studies of fungi associated with coprolites (e.g., Preussia, Sordaria, and Podospora) have demonstrated that coprophilous fungal spores, usually derived from sediment cores and coincident with pollen sequences, can be used to evaluate herbivore presence and abundance (Gill et al. 2012). Preussia spores, as a proxy for megafaunal biomass, have been used to infer changes in overall populations for numerous now-extinct megafaunal species in island and continental regions across the globe. For example, Gill and colleagues (2012) employ a multiproxy analysis of dung fungus spores that incorporates paleoecological and radiocarbon records to contextualize megaherbivore extinction processes around Silver Lake, Ohio. These various lines of evidence, which include AMS radiocarbon dates, calcium and strontium concentrations derived from X-ray fluorescence spectroscopy (XRF), pollen, charcoal, and sedimentary records, provide fine-grained insights into the interactions between megafauna, climate, and vegetation (Gill et al. 2012). A study of Preussia abundance in New Zealand demonstrated that this technique, which had previously been applied to mammalian megafauna in continental regions, was similarly effective for investigating past abundances of avian megafauna within island contexts (Wood et al. 2011). This technique has proven controversial, however, with species identifications and accumulation rates under varying hydrological conditions all subject to debate (Johnson et al. 2015, Dodson and Field 2018). Nevertheless, the introduction of fecal biomarker analysis, when combined with other paleoenvironmental indicators such as pollen and sedimentary DNA, may in future support inferences about megafaunal population abundance from sedimentary records.

## Linking past and present through new levels of detail

The suite of scientific methods available for studying megafaunal extinctions continues to expand and be refined, providing new opportunities to elucidate extinction processes operating on particular taxa and at individual localities, and enabling high-resolution investigations of the vulnerability or resilience of different species in the face of climate shifts and human intervention. These methods can clarify extinction chronology, taxonomy, dietary preferences, ecological relationships, environmental pressures, and ranging behaviors of diverse taxa that have often been unproductively grouped as simply, megafauna. In addition to highlighting the site- and species-specific variation in extinction causes and chronologies, these methods are generating more robust data sets that can also be used for large-scale modeling endeavors. Although methodological innovation and refinement can help inform paleoecological and taphonomic reconstruction, researchers will still need to rely on discontinuous or biased lines of evidence, each of which reflect a variety of contributing agents, site histories, and preservation conditions. Learning to deal with these issues as new methodological tools are applied poses new challenges for contemporary megafauna researchers. These limitations call for continuing dialogue between disciplines and the integration of complementary approaches.

Several recently published analyses highlight the interpretive power of combining cutting-edge, multidisciplinary methods, particularly in providing fine-grained ecological context around the disappearance of individual taxa from particular regions. Immel and colleagues (2015) used AMS radiocarbon dating, mtDNA, and stable isotope analysis to examine the disappearance of extinct giant deer (Megaloceros giganteus) from Central Europe. Through this integrative approach, they not only established a later date for the last known appearance of giant deer but also identified overlapping niches between giant deer, red deer, and reindeer as one possible cause of Megaloceros disappearance. Similarly, Terlato and colleagues (2019) combined chronometric, isotopic, and taphonomic analysis to push the last known appearance date of the European cave bear (Ursus spelaeus) to circa 24,200–23,000 BPE and suggest that cave bear feeding preferences did not change significantly over time. This, as well as taphonomic evidence for human–­cave-bear interaction, paints a complex picture of European cave bear extinction triggered by climate-driven niche contraction and human hunting pressure. A third example is the combined radiocarbon, stable isotope, DNA, and morphological analysis of Elasmotherium sibiricum, a giant rhinoceros also known as the Siberian unicorn, which has provided myriad new insights into the chronology and ecology of this species (Kosintsev et al. 2019). Previously thought to have gone extinct circa 200,000 years ago, new AMS dates suggest that this species persisted in Eastern Europe and Central Asia until at least 39,000 years ago, whereas DNA sequencing demonstrates that Elasmotheriinae diverged from Rhinocerotinae (the subfamily containing all living rhinoceros species) in the Eocene. Stable isotope and morphological analysis of E. sibiricum remains indicate that this species was adapted to dry steppe environments and had a highly specialized diet, factors that may have contributed to its eventual extinction (Kosintsev et al. 2019).

The more detail we are able to obtain in past analyses of megafaunal extinctions, the more we will be able to use archaeology and paleontology as a practical source of knowledge for modern conservation efforts and policy decisions. These new methods will also be important for understanding the long-term impact of extinctions on ecological processes and diversity in other groups of plants and animals. Most extant species evolved coevally with and ecologically connected to megafauna, and therefore, many are likely to be adapted either to megafauna themselves or to the environmental conditions that they created. Consequently, loss of these animals may have had large impacts on the abundance, life history, and survival of many other species. For example, the loss of species such as dung beetles and scavenging birds following giant herbivore extinctions likely also produced their own cascading ecosystem effects (Galetti et al. 2018).

Trophic rewilding efforts, which are intended to introduce large animals to specific environments to restore top-down trophic effects and nutrient flow, reverse trophic cascades, and return ecosystems to states of self-regulating biodiversity, rely on long-term and localized understandings of megafaunal positions within a given ecosystem. Louys and colleagues (2014) were able to draw on detailed paleontological histories of megafauna in the tropical Asia–Pacific region to inform current ecosystem restoration concerns. They evaluated the ranges and ecological roles of nine megafaunal taxa during the Pleistocene to assess their viability for rewilding efforts, including species translocations, reintroductions, or range expansions. By constructing a conservation translocation matrix, which allowed them to evaluate the degree of risk, benefit, and feasibility involved in rewilding each taxon, they found that orangutans, tapirs, and Tasmanian devils were the most well-suited to rewilding and ecosystem restoration endeavors in tropical Asia–Pacific. Although such studies demonstrate the potential for paleontological insights to contribute to modern conservation, they also highlight the need for increased applications of advanced laboratory methods to local-scale megafauna studies in order to set realistic goals for environmental restoration.

Beyond rewilding, detailed understanding of the main threats facing different megafaunal taxa in different contexts also allow the identification of the greatest risks facing particular animals today. For example, Roberts and colleagues (2014) when analyzing a long record of megafaunal stability in India over the past 200,000 years, argued that the biogeographic mosaic of India enabled species to move to similar ecosystems in different parts of the Indian subcontinent, even as climate and human pressures may have imposed themselves in a local context. This connectivity facilitated long-term overall survival of taxa in India despite local extirpation, but is threatened by growing infrastructure projects, agricultural expansion, and urban development. This long-term view complemented existing research that highlighted the maintenance of corridors between preferred environments, rather than the simple maintenance of an environment itself, for the persistence of various megafaunal taxa in the region (Roberts et al. 2014). The application of diverse methodologies to such records will only further the resolution of attempts to connect insights from the past to concerns in the present, enabling archaeology and paleontology to make a meaningful contribution to species and habitat protection or, where desired, reestablishment.

## Notes

### Author Biographical

Jillian A. Swift (jillian.swift@bishopmuseum.org), Michael Petraglia, Nicole Boivin, and Patrick Roberts are affiliated with the Department of Archaeology at the Max Planck Institute for the Science of Human History, in Jena, Germany. Jillian A. Swift is affiliated with the Anthropology Department of Bernice Pauahi Bishop Museum, in Honolulu, Hawai’i. Michael Bunce is affiliated with the Trace and Environmental DNA Laboratory, in the School of Molecular and Life Sciences, at Curtin University, in Bentley, Western Australia, in Australia. Joe Dortch is affiliated with the Centre for Rock Art Research and Management, at the University of Western Australia, in Perth, Australia. Kristina Douglass is affiliated with the Department of Anthropology and with the Institutes for Energy and the Environment at The Pennsylvania State University, in State College, Pennsylvania. J. Tyler Faith is affiliated with the Natural History Museum of Utah and with the Department of Anthropology at the University of Utah, in Salt Lake City, Utah. James A. Fellows Yates is affiliated with the Department of Archaeogenetics at the Max Planck Institute for the Science of Human History, in Jena, Germany. Judith Field is affiliated with the School of Biological, Earth, and Environmental Science at the University of New South Wales, in Sydney, Australia. Simon G. Haberle is affiliated with the College of Asia and the Pacific and the School of Culture, History, and Language at Australian National University, in Canberra, Australia. Simon G. Haberle and Chris N. Johnson are affiliated with the Australian Research Council Centre of Excellence in Australian Biodiversity and Heritage, in Wollongong, New South Wales, Australia. Eileen Jacob is affiliated with the Research Laboratory for Archaeology and the History of Art at the University of Oxford, in Oxford, England. Chris N. Johnson is affiliated with the School of Natural Sciences at the University of Tasmania, in Hobart, Australia. Emily Lindsey and Alexis M. Mychajliw are affiliated with the La Brea Tar Pits and Museum, part of the Natural History Museum of Los Angeles County, in Los Angeles, California. Eline D. Lorenzen is affiliated with the Natural History Museum of Denmark, at the University of Copenhagen, in Copenhagen, Denmark. Julien Louys is affiliated with the Australian Research Center for Human Evolution, Environmental Futures Research Institute at Griffith University, in Brisbane, Queensland, Australia. Gifford Miller is affiliated with the INSTAAR and the Department of Geological Sciences at the University of Colorado, in Boulder. Viviane Slon is affiliated with the Department of Evolutionary Genetics at the Max Planck Institute for Evolutionary Anthropology, in Leipzig, Germany. Natalia A. Villavicencio is affiliated with the Departamento de Ecología, in the Facultad de Ciencias Biológicas, at Pontificia Universidad Católica de Chile, in Santiago, Chile. Natalia A. Villavicencio is affiliated with the Instituto de Ecología and Biodiversidad, in Santiago, Chile. Michael R. Waters is affiliated with the Center for the Study of the First Americans, in the Department of Anthropology at Texas A&M University, in College Station, Texas. Frido Welker is affiliated with the Evolutionary Genomics Section of the GLOBE Institute, at the University of Copenhagen, in Copenhagen, Denmark, and with the Department of Human Evolution at the Max Planck Institute for Evolutionary Anthropology, in Leipzig, Germany. Rachel Wood is affiliated with the Research School of Earth Sciences at Australian National University, in Canberra, Australia.
